# An incidental finding of a hemoglobin E variant in a diabetic patient with an abnormal glycated hemoglobin level: a case report

**DOI:** 10.1186/s13256-024-04518-y

**Published:** 2024-06-15

**Authors:** Rashmi Karki, Samir Lamichhane, Runa Jha, Rekha Manandhar

**Affiliations:** 1 National Public Health Laboratory (NPHL), Kathmandu, Nepal; 2 Department of Clinical Pharmacology, Maharajgunj Medical Campus (MMC), Institute of Medicine (IOM), Tribhuvan University (TU), Kathmandu, Nepal

**Keywords:** Hemoglobin E (HbE), Glycated hemoglobin (HbA1c), Case report

## Abstract

**Background:**

Glycated hemoglobin is a well-known marker for evaluating long-term glycemic control. However, the accuracy of glycated hemoglobin measurement can be affected by the presence of hemoglobin variants, which makes the determination and interpretation of glycated hemoglobin values in terms of glycemic control not only difficult but also misleading. Here we present the first ever case of a patient with type 2 diabetes with hemoglobin E from Nepal, diagnosed incidentally because of spurious glycated hemoglobin levels.

**Case presentation:**

A 45-year-old Hindu Mongolian female with a history of type 2 diabetes for around 9 years but not very compliant with follow-ups was referred to our facility for plasma fasting and postprandial blood glucose levels and glycated hemoglobin. Fasting and postprandial blood sugars were found to be high. A consistent very low glycated hemoglobin by two different high-performance liquid chromatography (HPLC) methods compelled us to call the patient for a detailed clinical history and for the records of investigations done in the past. The patient has been a known case of type 2 diabetes for around 9 years and presented irregularly for follow-up visits. Around 4 years ago, she presented to a healthcare facility with fatigue, severe headaches, pain in the abdomen, discomfort, and dizziness for a couple of months, where she was shown to have high blood glucose. She was referred to a tertiary-level hospital in Kathmandu, where she was prescribed metformin 500 mg once daily (OD). Due to her abnormal hemoglobin A1c reports, she was then sent to the National Public Health Laboratory for repeat investigations. Her blood and urine investigations were sent. Complete blood count findings revealed high red blood cell and white blood cell counts, a low mean corpuscular volume, and a high red cell distribution width-coefficient of variation. Other parameters, including serum electrolytes, renal function tests, liver function tests, and urine routine examinations, were within normal limits. A peripheral blood smear revealed microcytic hypochromic red cells with some target cells. Hemoglobin electrophoresis showed a very high percentage of hemoglobin E, a very low percentage of hemoglobin A2, and normal proportions of hemoglobin A and hemoglobin F. A diagnosis of homozygous hemoglobin E was made, and family screening was advised.

**Conclusions:**

Clinicians should be aware of the limitations of glycated hemoglobin estimation by ion exchange high-performance liquid chromatography in patients with hemoglobin E and other hemoglobin variants. If the clinical impression and glycated hemoglobin test results do not match, glycated hemoglobin values should be determined with a second method based on a different principle, and glycemic status should be confirmed through alternative investigations, preferably those that are not influenced by the presence of hemoglobin variants (for example, boronate affinity chromatography, fructosamine test, glycated albumin test, the oral glucose tolerance test, continuous glucose monitoring, etc.). Consistent or even doubtful results should also raise the suspicion of a hemoglobin variant, which should be confirmed through further evaluation and investigations.

## Introduction

Glycated hemoglobin (HbA1c), initially identified as “unusual” hemoglobin in patients with diabetes over half a century ago [1], has now become a well-known marker of long-term glycemic control in individuals with diabetes mellitus [2], reflecting an average blood glucose level over a period of around 3 months. Glycemic control being an important factor for the progression to long-term complications, HbA1c strongly correlates with the risk of developing chronic complications associated with diabetes as well [3]; hence, it has become not only a diagnostic tool but also a screening tool for individuals at risk of diabetes [4]. However, the accuracy of HbA1c measurement can be affected by various factors such as erythropoiesis, glycation, erythrocyte destruction, and the presence of hemoglobin (Hb) variants [5], which makes the determination and interpretation of HbA1c values in terms of glycemic control not only difficult but also misleading [6]. Here, we present a case of a variant of Hb, the HbE, diagnosed incidentally in a patient with type 2 diabetes mellitus suspected initially because of repeated abnormal HbA1c levels detected with an ion-exchange high-performance liquid chromatography (HPLC) and hence subjected to further investigations by Hb capillary electrophoresis to confirm the diagnosis. To our knowledge, this is the first ever incidentally diagnosed case of HbE in a patient with type 2 diabetes from Nepal, all because of spurious HbA1c levels.

## Case presentation

A 45-year-old Hindu Mongolian female, with a history of type 2 diabetes for 9 years and under medication for the last 4 months, visited the National Public Health Laboratory (NPHL), Teku, to test for plasma fasting and postprandial blood glucose levels and HbA1c.

**Fig. 1:**
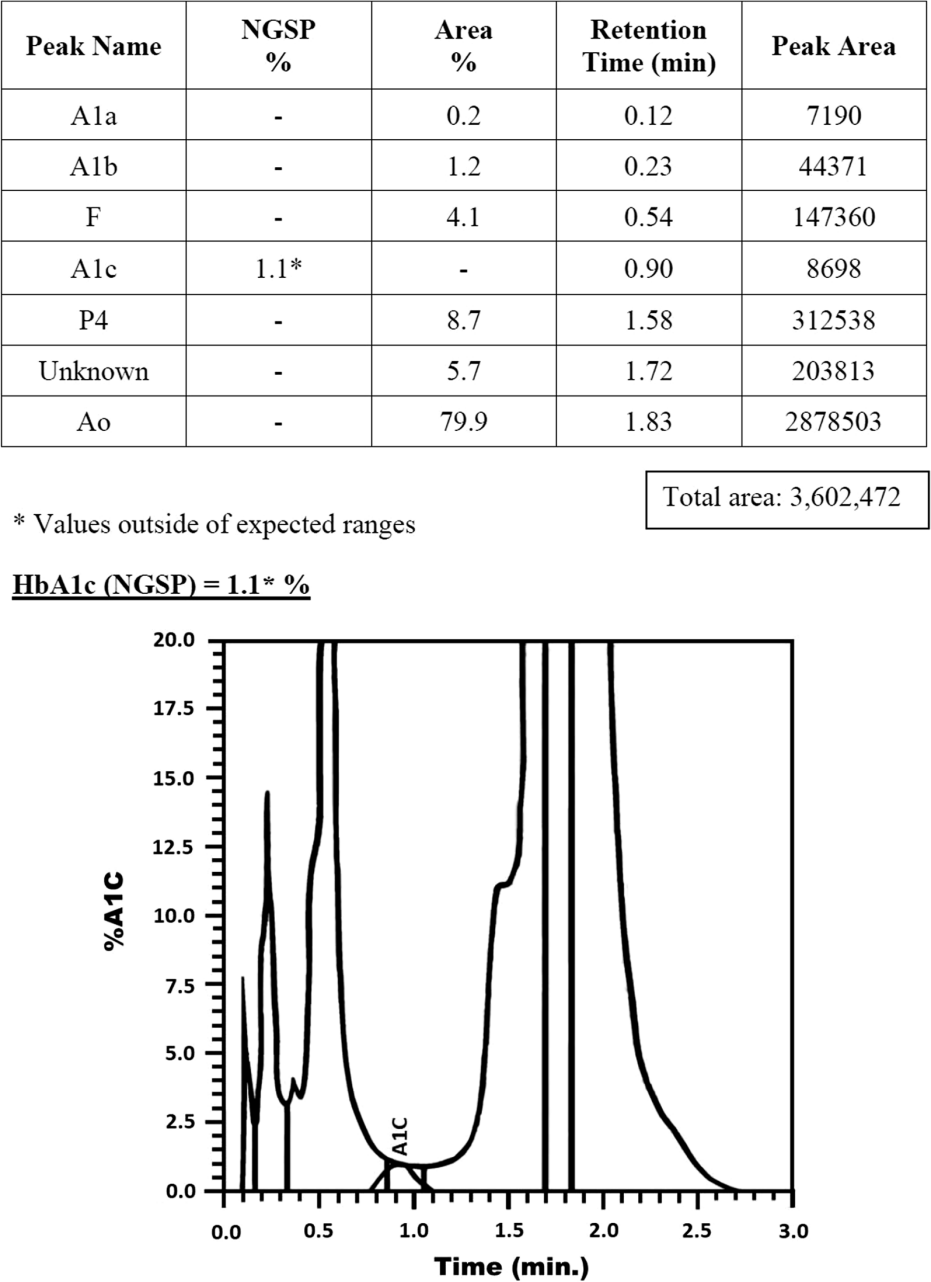
HbA1c detection by HPLC

We tested HbA1c by high-performance liquid chromatography (HPLC) Biorad VARIANT II, which showed HbA1c to be only 1.1% (Fig. 1). The control test run for the day was within range. Fasting and postprandial blood sugars were found to be 173 mg/dL and 280 mg/dL, respectively. The patient sample was rechecked with HPLC TOSO 7234X, which showed HbA1c of 2.1%. In view of this, the patient was contacted and advised to have a complete blood count (CBC) and hemoglobin electrophoresis. We took a detailed history of the patient and requested the records of investigations done in the past.

According to the patient, she was apparently well around 9 years ago (the patient herself was not sure about the exact date) when she started developing increased urination, increased thirst, weakness, and occasional dizziness for a couple of months. She then visited a local health care facility near her hometown, where she was examined and sent for some blood and urine investigations (she has misplaced the lab reports, or probably lost them, according to her). According to her, she was told that she has high blood sugar levels and was advised to make some lifestyle modifications, such as dietary changes and increasing physical activities. She was also prescribed medication (she has no records of the medications prescribed then) and was asked to follow up in the next 3 months. Thereafter, she was doing fine with no complaints for a couple of years, and she never followed up with any healthcare facility until around 3–4 years ago (May 2020), when she presented herself to a healthcare facility in Kathmandu city with complaints of fatigue, severe headaches, pain and discomfort in the abdomen, and occasional dizziness and a feeling of lightheadedness for a couple of months. She also complained of weakness and decreased tolerance for physical activity. Physical examination was unremarkable, and laboratory investigations revealed high blood glucose and serum triglyceride levels (fasting blood glucose: 175 mg/dL; postprandial blood glucose: 193 mg/dL; serum triglyceride: 290 mg/dL). Other investigations were within normal limits (Table 1).

**Table 1:** Laboratory findings with normal reference ranges for the blood investigations (done at various times)

S. No.	Parameters	Patient’s reading (reference range)
	*May 2020*	
1	Fasting blood glucose	**175** (70–99) mg/dL
2	Postprandial blood glucose	**193** (< 140) mg/dL
3	Total cholesterol	163 (< 200) mg/dL
4	Low-density lipoprotein (LDL) cholesterol	61 (< 100) mg/dL
5	High-density lipoprotein (HDL) cholesterol	44 (< 40: low; 40–59: borderline; > 60: considered protective) mg/dL
6	Triglyceride	**290** (50–150) mg/dL
	*1 January 2021*	
7	Total leucocyte count (TLC)	**11,660** (4000–11,000) cells/mcL
8	Differential leucocyte count (DLC)	N = **76** (40–60), L = **17** (20–40), M = 5 (2–8), E = 1 (1–4), B = 1 (0.5–1)%
9	Erythrocyte sedimentation rate (ESR)	10 (0–20) mm first hour
10	Platelet count	2,80,000 (150,000–450,000) cells/mcL
11	Hematocrit (Hct)	**37.7** (38.3–48.6) %
12	Mean corpuscular volume (MCV)	**64.4** (80–100) fL
13	Mean hemoglobin concentration (MCH)	**21.9** (27–32) pg
14	Mean corpuscular hemoglobin concentration (MCHC)	34 (32–36) gm% or g/dL
15	Red cell distribution width-standard deviation (RDW-SD)	**36.1** (40–55) fL
16	Red cell distribution width-coefficient of variation (RDW-CV)	**17.3** (11–15) %
17	Postprandial blood glucose	**294** (< 140) mg/dL
18	HbA1c	**2.1** (< 5.7)%
19	Urea	16.8 (6–20) mg/dL
20	Creatinine	(0.6–1.1) mg/dL
21	Sodium	136 (136–145) mEq/L
22	Potassium	4.27 (3.5–5.1) mEq/L
23	Total bilirubin	0.73 (0.2–1.2) mg/dL
24	Direct bilirubin	0.25 (0.0–0.4) mg/dL
25	Alanine transaminase (ALT)	17.9 (< 35) IU/L
26	Aspartate transaminase (AST)	13 (< 35 IU/L)
27	Alkaline phosphatase (ALP)	103 (38–154) U/L
28	Uric acid	5.5 (2.5–6.2) mg/dL
29	Phosphorous	3.24 (2.7–4.5) mg/dL
30	Calcium	**10.48** (8.6–10) mg/dL
31	Total protein	7.8 (6–8) g/dL
32	Albumin	4.8 (3.5–5.5) g/dL
33	Thyroid stimulating hormone (TSH)	2.56 (0.5–5) mIU/L
	*21 January 2021; at NPHL*	
34	Hemoglobin (Hb)	13.6 (12–16) g/dL
35	Red blood cell (RBC) count	**6.2 × 10**^**6**^ (3.5–5.0 × 10^6^) cells/mcL
36	Total leucocyte count (TLC)	**13,700** (4000–11,000) cells/mcL
37	Differential leucocyte count (DLC)	*N* = **77** (40–60), L = **18** (20–40), M = 4 (2–8), E = 1 (1–4), B = 0 (0.5–1)%
38	Platelet count	2.8 × 10^5^ (150,000–450,000) cells/mcL
39	Packed cell volume (PCV)	42.1 (38.3–48.6%)
40	Mean corpuscular volume (MCV)	**67** (80–100) fL
41	Mean hemoglobin concentration (MCH)	**21.7** (27–32) pg
42	Mean corpuscular hemoglobin concentration (MCHC)	32.3 (32–36) g/dL
43	Red cell distribution width–coefficient of variation (RDW-CV)	**17.9** (11.0–15.0)%
44	Total cholesterol	163 (< 200 mg/dL)
45	Triglycerides	**290** (50–150) mg/dL
46	High-density lipoprotein (HDL) cholesterol	44 (< 40: low; 40–59: borderline; > 60: considered protective) mg/dL
47	Very-low-density lipoprotein (VLDL) cholesterol	**58** (2–40) mg/dL
48	Low-density lipoprotein (LDL) cholesterol	61 (< 100) mg/dL
49	Urea	16.8 (6–20) mg/dL
50	Creatinine	0.6 (0.6–1.1) mg/dL
51	Uric acid	5.8 (2.5–6.2) mg/dL
52	Sodium	**134.8** (136–145) mEq/L
53	Potassium	4.5 (3.5 -5.1) mEq/L
54	Total bilirubin	**1.3** (0.2–1.2) mg/dL
55	Direct bilirubin	0.2 (0.0–0.4) mg/dL
56	Aspartate transaminase (AST)	14.8 (< 35) IU/L
57	Alanine transaminase (ALT)	10.4 (< 35) IU/L
58	Serum iron	88.7 (50–170) mcg/dL
59	Unsaturated iron binding capacity (UIBC)	276.4 (111–343) mcg/dL
60	Total iron binding capacity (TIBC)	365 (300–450) mcg/dL
61	Calcium	9.3 (8.6–10) mg/dL
62	Phosphorus	3.0 (2.7–4.5) mg/dL
63	Creatine kinase	35.0 (34–145) IU/L
64	Lactate dehydrogenase (LDH)	137.5 (< 400) U/L
65	Total protein	**8.2** (6–8) g/dL
66	Albumin	4.8 (3.5–5.5) g/dL
67	Albumin/globulin ratio	1 (1.0–1.8)
68	Alkaline phosphatase (ALP)	92.3 (38–154) U/L

The values in bold indicate that the parameter is out of range

With these reports, she was referred to a tertiary-level hospital in Kathmandu. On 1 January 2021, she visited Shree Birendra Hospital (a tertiary-level hospital in Kathmandu), where she was further evaluated. At this time, she also admitted having a history of a slight decrease in vision for the last few months and was evaluated for any eye findings. Apart from a slightly presbyopic finding, everything else was normal. Intraocular pressure was within normal limits. There were no findings suggestive of diabetic retinopathy. The fundus examination was normal. Further lab workup findings revealed the following: TLC: 11,660 cells/mcL; Differential Count (DC): *Neutrophils* = 76, Lymphocytes = 17, Monocytes = 5, Eosinophils = 1, Basophils = 1; MCV: 64.4 fL; MCH: 21.9 pg; RDW-SD: 36.1 fL; RDW-CV: 17.3 fL; postprandial (PP) blood glucose (two hours after taking a meal): 294 mg/dL; and HbA1c: 2.1%. Ultrasonography (USG) of the abdomen and pelvis showed a bulky uterus. All other investigations were within normal limits (Table 1).

She was prescribed metformin 500 mg OD and advised to make lifestyle modifications once again. She was then referred to the National Public Health Laboratory (NPHL), the central reference laboratory in Nepal, for repeat investigations to confirm the abnormal HbA1c reports and for all other relevant investigations. On arrival at NPHL (21 January 2021), she was thoroughly interviewed for a detailed medical history. She was a nonvegetarian, and she did not smoke tobacco or consume alcohol. She had never received a blood transfusion before. Apart from the metformin prescribed earlier, she was not on any other medications. There was no family history of low hemoglobin levels or blood transfusions. There was no other significant medical history in any of her family members, as far as the patient could remember. She gave a history of iron supplementation a long time ago (around 9 years ago when she first presented to a healthcare facility) for around 3 months but not thereafter. She denies any history of any means of blood loss. She has been married for around 25 years and has two kids (elder son: 23 years old, younger daughter: 21 years old). Physical examination was unremarkable and provided the following data: height: 5 feet; weight: 68 kg; body mass index (BMI): 29.3. Laboratory investigations are as follows:

CBC findings revealed hemoglobin of 13.6 g/dL, RBC of 6.2 × 10^6^ cells/mcL, total leucocyte counts of 13,700 cells/mcL (*N* = 77, L = 18, M = 4, E = 1, B = 0), platelet count of 2.8 × 10^5^ cells/mcL, and packed cell volume (PCV) of 42.1%. Red cell indices included mean corpuscular volume (MCV), mean hemoglobin concentration (MCH), mean corpuscular hemoglobin concentration (MCHC), and RDW-CV%, which were found to be 67 fL, 21.7 pg, 32.3 g/dL, and 17.9%, respectively. All other investigations were within normal limits (Table 1).

The ultrasonography (USG) report of the abdomen and pelvis revealed a bulky uterus and was otherwise unremarkable.

**Fig. 2:**
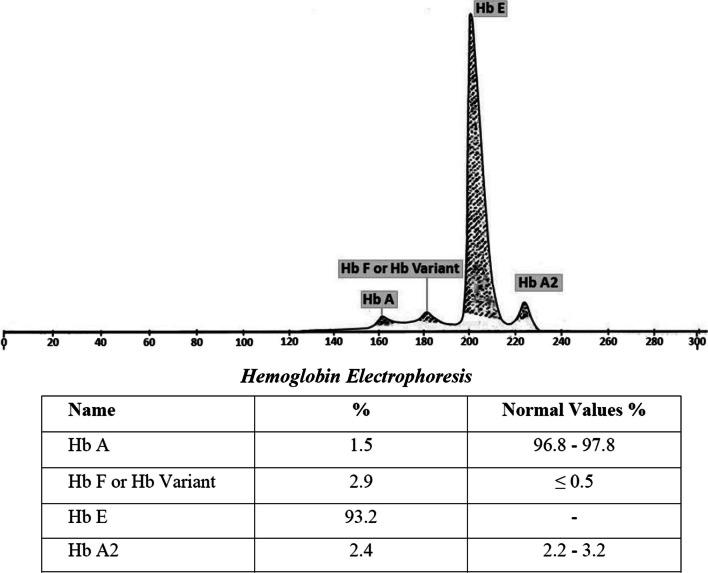
Detection of HbE by capillary electrophoresis

Hb electrophoresis was performed in the SEBIA MINICAP FLEX PIERCING electrophoretogram, which showed 93.2% HbE, 2.4% HbA2, 2.9% HbF, and 1.5% HbA (Fig. 2). A peripheral blood smear revealed microcytic hypochromic red cells with some target cells. White blood cell (WBC) and platelet morphology seem to have no abnormalities.

On the basis od these findings, a diagnosis of homozygous HbE was made, and family screening was advised.

## Discussion and conclusion

Glycated hemoglobin (HbA1c), an effective and objective retrospective marker reflecting an average blood glucose level over a period of around 3 months, has now become a well-known indicator of long-term glycemic control in individuals with diabetes mellitus. However, there are various factors that may influence and falsely alter the level of HbA1c and its measurement and hence need to be considered in patients with abnormal readings.

HbA1c levels seem to inversely correlate with the rate of erythropoiesis, and hence factors that decrease the rate of erythropoiesis (including iron, vitamin B_12_, and folate deficiency) and/or increase erythrocyte life span (for example, due to splenectomy) falsely increase the level of HbA1c. Conversely, administration of erythropoietin, iron, and vitamin B_12_ and conditions associated with reticulocytosis and decreased erythrocyte lifespan (for example, splenomegaly or even pregnancy) tend to falsely decrease the level of HbA1c [1, 7]. There has been evidence of a false increase in HbA1c in cases of alcoholism and in patients with chronic kidney disease (CKD), as well, possibly through the same mechanism (alcohol interferes with folate metabolism, and patients with CKD have decreased erythropoietin levels) [1, 7, 8]. Several other conditions, for example, hyperbilirubinemia, carbamylated hemoglobin, chronic opiate use, etc., are also seen to be associated with a high HbA1c level [1]. However, chronic liver disease, rheumatoid arthritis, hypertriglyceridemia, and even the use of drugs such as ribavirin and dapsone have been shown to be associated with decreased HbA1c [1, 7, 9, 10]. Genetic or chemical alterations in hemoglobin undoubtedly have some associations with HbA1c, and hence certain hemoglobinopathies, including the HbE disease, the presence of HbF, and methemoglobinemia, may also alter the level of HbA1c [1, 7, 11]. Here we discuss the presence of HbE and the misleading value of HbA1c levels.

Hemoglobin E, a variant hemoglobin, is characterized by a mutation in the β globin gene (*HBB* gene) causing substitution of glutamic acid for lysine at position 26 of the β globin chain, resulting in a heterogeneous group of disorders whose phenotypes range from asymptomatic to severe disease [12, 13]. HbE trait and HbEE are mild disorders, while a combination of HbE with other forms of hemoglobinopathies does exist that can have a markedly different and more serious clinical course, producing a wide range of clinical syndromes of varying severity [13, 14]. The heterozygous form of HbE is usually characterized by minimal red cell morphological abnormalities and normal red cell indices, while homozygotes for HbE can have red cells with significant morphological abnormalities, including increased numbers of target cells, and can present with mild microcytic hypochromic anemia [14].

Despite advances in the standardization of methods for glycohemoglobins, including HbA1c, an increasing number of hemoglobinopathies have been shown to interfere with the accurate measurements and determination of these glycohemoglobins. Even the most commonly used methods, that is, the HPLC methods for HbA1c determination, lacked the resolution necessary to differentiate hemoglobin variants [6]. The demonstration of additional peaks in the chromatograms and either too low or too high values of HbA1c has been shown as compared with the nondiabetic reference range in different types of hemoglobinopathies [15].

Patients homozygous for HbE, that is, those receiving mutated genes from both parents, have a very low HbA level, with around 80% of Hb being HbE itself. The mutation associated with HbE tends to alter the ionic charges on Hb and hence interferes with the measurement of HbA1c via the ion-exchange HPLC method, especially in homozygous cases [16, 17]. Unique mutation(s) on the N-terminal of β-globin in some hemoglobinopathies such as the Hb Graz and the Hb Long Island variants also seem to cause inappropriately high and low apparent HbA1c titers via HPLC methods. However, estimations with the boronate affinity technique and the immunoassay technique seem to be unaffected. The boronate affinity method has shown values in an acceptable and clinically reasonable range for all hemoglobin variants, as evidenced by several studies [18, 19]. In fact, affinity methods have already been suggested as an acceptable and more useful method for reflecting glycemic control because they mainly measure glycohemoglobin regardless of the glycation site and hence may be clinically more accurate [15]. Studies have shown that results from the HbA1c immunoassays were also comparable to those from HPLC assays, showing good correlation, appropriate precision, and low bias [20]. Immunoassays utilizing various antibodies raised against specific epitopes of hemoglobin, for example, the Amadori product of glucose plus the first eight amino acids on the N-terminal end of the beta chain of hemoglobin, and many more, have shown good correlation with established methods for estimating glycohemoglobin [21]. However, as the quality of an immunoassay typically depends on the specificity of the antibody to the specific epitope on HbA, specific mutations altering the common epitopes used for the assays will hinder the accuracy of the test. One such example includes the hemoglobin variant with mutations affecting or altering the epitope at the N-terminal chain. The mutation seems to affect the ability of the monoclonal antibody that is used in the assay to detect hemoglobin [22]. Some uncommonly occurring variants that span the commonly used epitope include HbE and HbD (Los Angeles), where mutations occur at β26 and β121, respectively. There are some other evidences/studies that have reported that immunoassays have been shown to produce false HbA1c results in certain Hb variants [23–26]. Hence, choosing a method where the antibody epitope does not span the specific area in the Hb variant is crucial. However, it is not practical or even feasible to produce several specific antibodies (according to the individual patient) at each facility, and hence understanding the effects of such hemoglobinopathies while estimating the glycohemoglobins is crucial. To be precise, the effect of various hemoglobinopathies on HbA1c measurements is highly method-dependent. So, it is always better to be correlated clinically, and whenever the HbA1c results do not fit the clinical picture, some additional peaks in HPLC chromatograms are displayed, or any such doubtful scenario has been presented, it should not be ignored, and further investigations are advisable. Glycemic status over a short period of time (1–3 weeks) can also be reflected by the fructosamine test. Some researchers have therefore recommended confirmation with the fructosamine test or the glycated albumin test as an alternative [14, 24, 27, 28]. Fructosamine results depend on the glycation of serum proteins and are not influenced by hemoglobin variants [19]. However, falsely low levels may occur in patients with hypoalbuminemia, for example, in patients with nephrotic syndrome or severe liver disease [29]. The glycated albumin test, reported as a percentage of total albumin, also reflects short-term glycemic status, typically over the preceding 2–3 weeks, and is not influenced by situations that falsely alter A1C levels [29, 30]. Moreover, the tests that rely purely on blood glucose levels, including the oral glucose tolerance test (OGTT) and even continuous glucose monitoring, could possibly be the ones that are least affected by various factors, as discussed earlier. The OGTT is advocated for screening and diagnosis, and self-monitoring of blood glucose levels is advised for management during pregnancy [29]. Continuous glucose monitoring for up to 5 days has also been shown to correlate well with HbA1c levels [29].

The mutations associated with hemoglobin E disease are primarily seen to be prevalent in the eastern half of the Indian subcontinent and throughout Southeast Asia [12, 23]. In 1954, Chernoff and colleagues first described that it has occurred in conjunction with β thalassemia, in which case it presents with a severe form of the disease known as the compound heterozygosity for hemoglobin E/β thalassemia [24], and since then several other cases have been reported from several parts of Southeast Asia [25–27, 31]. Cases have been reported from some parts of Nepal, as well, but to our knowledge, this case report is the first ever report of an incidentally diagnosed HbE variant in a patient with type 2 diabetes mellitus in Nepal.

Several studies have evidenced and reported that various hemoglobinopathies, including HbE disease, interfere with accurate measurements of glycosylated hemoglobin, including HbA1c. A study conducted on the prevalence of hemoglobin variants and their effect on HbA1c measurement among the indigenous population of north Bengal showed Hb variants to have a significant effect on HbA1c measurement [32]. A clinically silent and very rare hemoglobinopathy, hemoglobin Himeji, has been reported in a Portuguese patient with diabetes with a discrepancy between fasting plasma glucose and HbA1c [33]. Yet another case series of two female Malay patients with HbJ, an Hb variant, showed persistently high HbA1c levels despite good glycemic control [34].

Because of the local occurrence of Hb variants and the ethnic origin of a given population, every individual laboratory must establish and validate its own assay method. Also, while managing patients with diabetes, knowledge of hemoglobinopathies influencing HbA1c determination methods is essential. Moreover, in populations with a high prevalence of hemoglobinopathies, hemoglobin typing should be considered basic information prior to HbA1c measurement, as suggested by some other studies, as well [35].

Hence, to conclude, clinicians should be aware of this limitation of HbA1c estimation by ion-exchange HPLC in patients with HbE and other Hb variants, though HPLC has been an important and one of the most commonly used modalities [26], among the several others such as immunoassay techniques, boronate affinity chromatography, etc., to detect it [18]. If the clinical impression and HbA1c test results do not match, then HbA1c values should be determined with a second method based on a different principle and confirmation of the glycemic status through alternative investigations. The boronate affinity method has been shown to be clinically reasonable for all hemoglobin variants. Similarly, the fructosamine test and the glycated albumin test could also be used as alternatives, as they are also not influenced by the presence of hemoglobin variants. The OGTT and continuous glucose monitoring can obviously be other reliable alternatives, though they have their own disadvantages, such as the fact that they will not reflect glycemic control over a longer period of time, as does the HbA1c, and need repeated measurements. Moreover, an abnormal HbA1c level in a diabetic patient or any other subject during routine evaluation or screening for diabetes could raise the suspicion of an Hb variant. Therefore, physicians and especially endocrinologists should take this fact into account and immediately seek further evaluation and investigations to confirm the diagnosis of Hb variants in such patients and advise the patients to screen their family members.

## Data Availability

Data and other materials can be made available if required.

## References

[1] Abbreviated Report of a WHO Consultation. Use of glycated haemoglobin (HbA1c) in the diagnosis of diabetes mellitus. 2011. 26158184

[2] Campbell L, Pepper T, Shipman K (2019). HbA1c: a review of non-glycaemic variables. J Clin Pathol.

[3] UK Prospective Diabetes Study (UKPDS) Group (1998). Intensive blood-glucose control with sulphonylureas or insulin compared with conventional treatment and risk of complications in patients with type 2 diabetes (UKPDS 33). Lancet.

[4] Lim WY (2018). Screening for diabetes with HbA1c: test performance of HbA1c compared to fasting plasma glucose among Chinese, Malay and Indian community residents in Singapore. Sci Rep.

[5] Nureslyna SI (2013). Percentage of haemoglobin variants detected during HbA1c analysis in hospital Kuala Lumpur. Malays J Med Health Sci.

[6] Schnedl WJ (2000). Evaluation of HbA1c determination methods in patients with hemoglobinopathies. Diabetes Care.

[7] Chen Z (2022). Interpretation of HbA1c lies at the intersection of analytical methodology, clinical biochemistry and hematology (review). Exp Ther Med.

[8] Bloomgarden Z, Handelsman Y (2018). How does CKD affect HbA1c?. J Diabetes.

[9] Sehrawat T (2018). Utility and limitations of glycated hemoglobin (HbA1c) in patients with liver cirrhosis as compared with oral glucose tolerance test for diagnosis of diabetes. Diabetes Ther.

[10] Lewis D (1983). Glycosylated haemoglobin in rheumatoid arthritis. Ann Rheum Dis.

[11] Rhea JM, Molinaro R (2014). Pathology consultation on HbA(1c) methods and interferences. Am J Clin Pathol.

[12] Bachir D, Galacteros F. Hemoglobin E disease. Orphanet Encyclopedia. 2004.

[13] Braunstein EM. Hemoglobin E disease. Merck Manual. 2017.

[14] Fucharoen S, Weatherall DJ (2012). The hemoglobin E thalassemias. Cold Spring Harb Perspect Med.

[15] Eckfeldt JH, Bruns DE (1997). Another step toward standardization of methods for measuring hemoglobin A1c. Clin Chem.

[16] Musalmah M (2000). Effect of hemoglobin E on glycosylated hemoglobin determinations using different commercial kits. Med J Malaysia.

[17] Chen Y (2014). Influence and analysis of seven kinds of hemoglobin variants on HbA1c detection. Zhonghua Yixue Zazhi.

[18] Little RR, Roberts WL (2009). A review of variant hemoglobins interfering with hemoglobin A1c measurement. J Diabetes Sci Technol.

[19] Paisooksantivatana K (2009). Influence of hemoglobin E on measurement of hemoglobin A1c by immunoassays. Diabetes Res Clin Pract.

[20] Guadalupe Vargas M (2020). Assessment of two glycated hemoglobin immunoassays. Endocrinol Diabetes Nutr (Engl Ed).

[21] John WG (1993). Enzyme immunoassay—a new technique for estimating hemoglobin A1c. Clin Chem.

[22] Watanabe T (1998). A nondiabetic case of hemoglobin variant (Hb Niigata) with inappropriately high and low HbA1c titers detected by different methods. Clin Chem.

[23] Weatherall DJ, Clegg JB (2001). Inherited haemoglobin disorders: an increasing global health problem. Bull World Health Organ.

[24] Chernoff AI (1956). Studies on hemoglobin E. I. The clinical, hematologic, and genetic characteristics of the hemoglobin E syndromes. J Lab Clin Med..

[25] Minnich V (1954). Mediterranean anemia: a study of thirty-two cases in Thailand. Blood.

[26] Yedla N, Kuchay MS, Mithal A (2015). Hemoglobin E disease and glycosylated hemoglobin. Indian J Endocrinol Metab.

[27] Olivieri NF, Pakbaz Z, Vichinsky E (2011). Hb E/beta-thalassaemia: a common and clinically diverse disorder. Indian J Med Res.

[28] Ribeiro RT, Macedo MP, Raposo JF (2016). HbA1c, fructosamine, and glycated albumin in the detection of dysglycaemic conditions. Curr Diabetes Rev.

[29] Mahabeer S. HbA1c measurement and assay interferences. Pathologists Lancet Laboratories, newsletter; 4th quarter. 2018.

[30] Freitas PAC, Ehlert LR, Camargo JL (2017). Glycated albumin: a potential biomarker in diabetes. Arch Endocrinol Metab.

[31] Premawardhena A (2005). Haemoglobin E beta thalassaemia in Sri Lanka. Lancet.

[32] Roy S (2022). Prevalence of hemoglobin variants and their effect on Hba1c measurement among the indigenous population of north Bengal attending a tertiary care hospital. Asian J Med Sci.

[33] Guedes V (2017). Hemoglobin Himeji and inconsistent hemoglobin A1c values: a case report. J Med Case Rep.

[34] Wan Nik W (2022). Significantly high HbA(1c) in diabetic patient with Hb J: Case Report. Oman Med J.

[35] Mitchai M (2021). Misleading HbA1c measurement in diabetic patients with hemoglobin variants. Med Sci (Basel).

